# Evaluation of the Anti-Mycobacterial and Anti-Inflammatory Activities of the New Cardiotonic Steroid γ-Benzylidene Digoxin-15 in Macrophage Models of Infection

**DOI:** 10.3390/microorganisms13020269

**Published:** 2025-01-25

**Authors:** Daniel Wilson A. Magalhães, Maria Gabriella S. Sidrônio, Noêmia N. A. Nogueira, Deyse Cristina Madruga Carvalho, Maria Eugênia G. de Freitas, Ericke Cardoso Oliveira, Gustavo F. de Frazao Lima, Demétrius A. M. de Araújo, Cristoforo Scavone, Thalisson Amorim de Souza, José Augusto F. P. Villar, Leandro A. Barbosa, Francisco Jaime Bezerra Mendonça-Junior, Valnês S. Rodrigues-Junior, Sandra Rodrigues-Mascarenhas

**Affiliations:** 1 Postgraduate Program in Physiological Sciences, Federal University of Paraíba (UFPB), João Pessoa 58051-900, PB, Brazil; daniel.magalhaes.biotec@gmail.com (D.W.A.M.); noemianielly.a@gmail.com (N.N.A.N.); 2 Postgraduate Program in Development and Technological Innovation in Medicines, Federal University of Paraíba (UFPB), João Pessoa 58051-900, PB, Brazil; gabriellassidronio@gmail.com; 3 Laboratory of Immunobiotechnology, Biotechnology Center, Federal University of Paraíba (UFPB), João Pessoa 58051-900, PB, Brazil; deysecmc@gmail.com (D.C.M.C.); sandra@cbiotec.ufpb.br (S.R.-M.); 4 Laboratory of Biotechnology in Microorganisms, Biotechnology Center, Federal University of Paraíba (UFPB), João Pessoa 58051-900, PB, Brazil; m.eugeniagouveia@gmail.com; 5 Laboratory of Cellular Biochemistry, Campus Centro-Oeste Dona Lindu, Federal University of São João Del-Rei, Divinópolis 35501-296, MG, Brazil; erickecardosooliveira01@gmail.com (E.C.O.); frazao39@gmail.com (G.F.d.F.L.); zevillar@ufsj.edu.br (J.A.F.P.V.); lbarbosa@ufsj.edu.br (L.A.B.); 6 Postgraduate Program in Biotechnology (Renorbio), Federal University of Paraíba (UFPB), João Pessoa 58051-900, PB, Brazil; demetrius@cbiotec.ufpb.br; 7 Laboratory of Neuropharmacology Research, Department of Pharmacology, Institute of Biomedical Sciences ICB-1, University of São Paulo, São Paulo 05508-900, SP, Brazil; cristoforo.scavone@gmail.com; 8 Postgraduate Program in Natural and Synthetic Bioactive Products, Department of Pharmaceutical Sciences, Federal University of Paraíba (UFPB), João Pessoa 58051-900, PB, Brazil; thalisson.amorim@ltf.ufpb.br; 9 Laboratory of Synthesis and Drug Delivery, Department of Biological Sciences, State University of Paraíba, João Pessoa 58071-160, PB, Brazil

**Keywords:** tberculosis, drug development, host-directed therapy, anti-inflammatory, benzylidene digoxin

## Abstract

Cardiotonic steroids modulate various aspects of the inflammatory response. The synthetic cardiotonic steroid γ-benzylidene digoxin 15 (BD-15), a digoxin derivative, has emerged as a promising candidate with potential immunomodulatory effects. However, its biological activity remains largely unexplored. This study investigated the anti-mycobacterial and anti-inflammatory effects of BD-15 in an in vitro macrophage infection model with *Mycobacterium* spp. Unlike digoxin, which showed significant toxicity at higher concentrations, BD-15 exhibited no cytotoxicity in RAW 264.7 cells (a murine macrophage cell line). Both compounds were evaluated in *Mycobacterium smegmatis*-infected RAW 264.7 cells, reducing bacterial burden without direct bactericidal activity. Additionally, both modulated pro-inflammatory cytokine levels, notably by decreasing tumor necrosis factor alpha (TNF-α) and interleukin-1 beta (IL-1β) levels. BD-15 specifically reduced NOD-, LRR-, and pyrin-domain-containing protein 3 (NLRP3) inflammasome expression and increased interleukin-10 (IL-10) production. Notably, BD-15 reduced colony-forming unit (CFU) counts in *Mycobacterium tuberculosis*-infected RAW 264.7 cells. Toxicity assays in HepG2 cells (a human liver cancer cell line) showed that BD-15 had minimal hepatotoxicity compared to digoxin, and both demonstrated negligible acute toxicity in an *Artemia salina* bioassay. These findings revealed the immunomodulatory effects of cardiotonic steroids in a bacterial infection model and highlighted BD-15 as a safer alternative to digoxin for therapeutic applications.

## 1. Introduction

Tuberculosis (TB) is a chronic infectious disease caused by *Mycobacterium tuberculosis*, an obligate aerobic bacterium that primarily infects the cells of the mononuclear phagocytic system. TB remains one of the deadliest infectious diseases in the world, and along with HIV/AIDS and malaria, it has had one of the most significant socioeconomic impacts on humanity [1,2]. In 2023, it was estimated that 10.8 million individuals contracted TB, resulting in approximately 1.25 million deaths, with 1.09 million HIV-negative individuals and 161.000 among individuals with HIV. This situation is exacerbated by the global spread of multidrug-resistant TB (MDR-TB) and extensively drug-resistant TB (XDR-TB) [3].

Drug resistance occurs when bacteria mutate and become less sensitive or completely resistant to antibiotics, leading to treatment failure, prolonged illness, and potential spread of resistant strains to others. The emergence and spread of antimicrobial resistance in various diseases, including TB, present a severe challenge to global public health. Resistance significantly affects both individuals and healthcare systems worldwide, as infections often require longer treatment with more expensive and potentially more toxic drugs [4,5].

The search for new and effective antibacterial drugs to combat TB remains a critical priority, particularly in the face of drug-resistant TB. In recent years, there has been an increasing focus on understanding and targeting host biological processes that contribute to TB pathogenesis. Host-directed therapy (HDT) is a promising therapeutic strategy that, rather than directly targeting the pathogen, aims to modulate the host immune responses and cellular processes. This approach creates an environment that enhances the body’s natural ability to fight infections [6,7]. This approach focuses on highly conserved host signaling pathways that are less prone to mutations, potentially making these therapies effective against diverse TB strains. This strategy contrasts with conventional antibiotics that directly target bacterial components, enabling the development of drug-resistant strains [6,7].

Cardiotonic steroids, commonly used in the treatment of congestive heart failure, have been shown to exhibit a range of biological activities, including antiviral [8], antitumor [9], and anti-inflammatory effects [10]. Our research group previously identified the cardiotonic steroid ouabain as an immunomodulatory agent, demonstrating its anti-inflammatory activity in both in vitro and in vivo models. At low concentrations, ouabain can modulate several inflammatory parameters, such as cell migration, vascular permeability, and production of pro-inflammatory cytokines [10,11,12,13,14,15,16]. Additionally, derivatives of cardiotonic steroids have also been shown to regulate immune response [17]. A digoxin (DG) derivative BD-8 (γ-benzylidene digoxin 8), for instance, modulates the immune response of macrophages by decreasing phagocytosis, nitric oxide production, and IL-1β levels through suppression of the p38-ERK/NF-κB signaling pathway [18]. Similarly, BD-21 (γ-benzylidene digoxin 21) has been shown to significantly reduce paw edema, inhibiting the expression of inducible nitric oxide synthase (iNOS) and lowering TNF-α levels [19]. These findings highlight the potential of novel cardiotonic steroids as immunomodulatory agents, while ongoing efforts to synthesize new compounds with chemical modifications show promise in enhancing their therapeutic index and reducing toxicity risks [20,21,22,23].

BD-15 is a novel benzylidene DG derivative synthesized by our research group, but its anti-inflammatory and host-mediated effects have not yet been studied in a bacterial infection model. Therefore, this study aimed to investigate the anti-mycobacterial and anti-inflammatory activities of BD-15 and DG in an in vitro model of mycobacterial macrophage infection.

## 2. Materials and Methods

### 2.1. Synthesis of γ-Benzylidene Digoxin 15 (BD-15)

**Figure 1:**
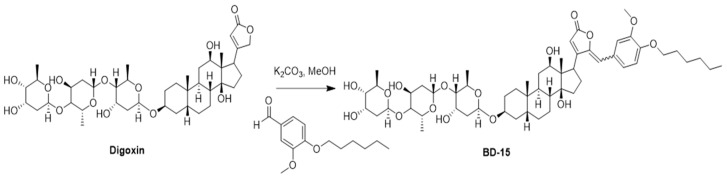
Reaction scheme for BD-15 synthesis. Aldehyde (1.8 mmol), DG (0.469 g, 0.6 mmol), and anhydrous K_2_CO_3_ (0.249 g, 1.8 mmol) were added to 60 mL of methanol in a round-bottom flask. After stirring for 6 h at 70 °C, the solvent was evaporated using a rotary evaporator. The crude product was diluted with 20 mL of water and extracted with hot ethyl acetate (3 × 30 mL). The organic layer was washed with brine, dried over anhydrous Na_2_SO_4_, and concentrated under a vacuum. The crude product was purified using silica column chromatography (CH_2_Cl_2_/MeOH 11:1). After purification, the product was diluted in THF, precipitated with hexane, and concentrated under reduced pressure to obtain the BD-15 derivative.

BD-15 was synthesized via a vinylogous aldol reaction between DG and the appropriate benzaldehyde. All compounds were characterized based on extensive analyses of ^1^H and ^13^C NMR, HRMS, and IR data (Supplementary Material). This synthesis was carried out at the Laboratory of Organic Synthesis and Nanostructures of the Federal University of São João del-Rei. The new cardiotonic steroid BD-15 has an additional aromatic group with a substituent ether [17]. The structure of BD-15 and the reaction scheme for its synthesis are shown in Figure 1.

The BD molecules were initially designed with the introduction of the benzylidene group to create steric hindrance in the Na/K-ATPase enzyme and consequently decrease the inhibition of this enzyme. However, it was observed that the elongation of the side chain induced activation of the alpha-3 subunit, which is associated with anti-inflammatory effects [17].

### 2.2. Bacteria and Cell Lines

*Mycobacterium smegmatis* mc^2^155 and *M. tuberculosis* H37Ra laboratory strains were maintained in Middlebrook 7H9 (Difco) media supplemented with Tween-80 (0.05%) and 10% oleic acid-albumin-dextrose-catalase (OADC, Becton Dickinson, Singapore). *M. smegmatis* and *M. tuberculosis* inocula were prepared as previously described [24]. The murine macrophage cell line RAW 264.7 and human hepatocellular carcinoma HepG2 cells were obtained from the Cell Bank of Rio de Janeiro and cultured in Dulbecco’s modified Eagle ’s medium (DMEM) supplemented with 10% inactivated fetal bovine serum (FBS) and 1% antibiotics (penicillin-streptomycin). Cells were maintained in culture flasks at 37 °C in a humidified atmosphere containing 5% CO_2_.

### 2.3. Evaluation of the Direct Effects of BD-15 and DG on RAW 264.7 Cell Viability

The cytotoxic effects of BD-15 and DG on RAW 264.7 cell viability were evaluated using an MTT (3-(4,5-dimethylthiazol-2-yl)-2,5-diphenyltetrazolium bromide) assay [25]. RAW 264.7 cells were seeded at 1 × 10^4^ cells/well (for 24 h incubation) or 5 × 10^3^ cells/well (for 48 h incubation) and incubated overnight to allow for adhesion. The culture medium was carefully aspirated and replaced with 100 μL of DMEM plus 100 μL of the test compound solutions, yielding final concentrations ranging from 0.01 to 10 µM (DMSO 0.5%, *v*/*v*). RAW 264.7 cells treated with 10% DMSO served as a positive control for cell death. After 24 or 48 h of exposure, cells were incubated with MTT (0.5 mg/mL) for 3 h. The resulting formazan crystals were dried and dissolved in DMSO, and the absorbance was measured at 570 nm using a microplate reader (EL800, BioTek, Winooski, VT, USA). The amount of precipitated purple formazan crystals was directly proportional to the number of metabolically active viable cells. The cell viability was calculated as follows: cell viability (%) = (sample absorbance/control absorbance) × 100. Data are expressed as the mean cell viability ± standard error of the mean of three to four independent experiments performed in triplicate.

### 2.4. M. smegmatis Susceptibility Investigation

The susceptibility of *M. smegmatis* mc^2^155 to BD-15 and DG was investigated using 96-well plates following previously established protocols [24]. BD-15 and DG were initially solubilized in DMSO at a concentration of 8 mM and then diluted in Middlebrook 7H9 plus OADC enrichment (Becton Dickinson) to achieve a concentration of 0.4 mM. Serial two-fold dilutions were performed in 96-well U-bottom polystyrene microplates over a concentration range of 200–1.56 µM. The final DMSO concentration in all the wells was 2.5%. Mycobacterial suspensions were cultivated and diluted in 7H9 medium at an optical density (OD_595nm_) of 0.001, and 100 µL was added to each well. Following incubation at 37 °C for 24 h, 30 µL of sterile resazurin solution (0.02%) was added to the plates, and the results were evaluated after 24 h. The effects on bacterial viability were considered as the drug concentration that prevented a color change from blue (resazurin) to pink (resorufin) [24].

### 2.5. Evaluation of the Therapeutic Potential of BD-15 and DG in M. smegmatis-Infected RAW 264.7 Cells

For the in vitro infection test, RAW 264.7 cells were seeded in 96-well culture plates at a density of 1 × 10^4^ cells/well in DMEM medium (with 10% FBS, without antibiotics) and incubated overnight at 37 °C with 5% CO_2_ [25]. Infection of RAW 264.7 cells with *M. smegmatis* mc^2^155 was carried out at a multiplicity of infection (MOI) of 1:1 for 2 h at 37 °C with 5% CO_2_. The medium containing non-internalized bacteria was removed, and infected cells were treated with the tested substances in DMEM: BD-15 (10, 1, 0.1, and 0.01 µM) and DG (10, 1, 0.1, and 0.01 µM). After 24 h, the supernatant was collected for cytokine analysis, the cells were lysed with 0.1% Triton X-100, and the resulting suspensions were serially diluted and plated on Luria-Bertani (LB) agar. Colony-forming units (CFUs) were determined after incubating the plates for 3 days at 37 °C. The CFU numbers are expressed as log_10_ (CFUs/well).

### 2.6. Quantification of Cytokine Production

IL-1β, IL-10, and TNF-α levels in the culture supernatant were determined using a sandwich-enzyme-linked immunosorbent assay, as described previously [18].

### 2.7. NLRP3 Evaluation by Flow Cytometry

After 24 h of culture, the cells were transferred to a U-bottom 96-well plate. They were then fixed and permeabilized for 30 min each. Following this, the cells were labeled with an unconjugated anti-NLRP3 antibody, according to the manufacturer’s instructions (BD Biosciences, San Jose, CA, USA). Finally, anti-rat IgG2a (PE) was added for NLRP3 analysis, and the cells were resuspended in PBS for evaluation using a flow cytometer (BD Accuri C6 Plus), as described previously [18].

### 2.8. Cytotoxicity Evaluation of BD-15 and DG on HepG2

Cytotoxicity was evaluated using the MTT method [26]. For this purpose, the HepG2 cell line was used. Cells were seeded at a density of 2 × 10^4^ cells/well in 96-well microtiter plates and incubated overnight to allow for adherence. The culture medium was replaced with 100 µL of DMEM plus 100 µL of test compound solutions (BD-15 and DG) at final concentrations ranging from 15 to 120 µM (1.5% DMSO, *v*/*v*). HepG2 cells treated with 20% DMSO served as a positive control for cell death. The cells were incubated with the test compounds for 24 h at 37 °C in a 5% CO_2_ atmosphere. After incubation, MTT solution (0.5 mg/mL) was added, and the cells were incubated for an additional 3 h. The resulting formazan crystals were dried at room temperature for 24 h and dissolved in DMSO. The absorbance was measured at 570 nm using a microplate reader (EL800, BioTek, USA). Cell viability was calculated as previously described in the RAW 264.7 cell viability assay section.

### 2.9. Artemia salina Toxicity Evaluation

*Artemia salina* (brine shrimp) survival was determined after incubation with BD-15 and DG following the method described by Solis et al. [27]. *A. salina* cysts were purchased from a local aquarium store and hatched (0.5 g cysts/L) in artificial seawater (35 g/L sea salt) supplemented with dried yeast (6 mg/L). Aeration was performed using an aquarium air pump connected to a line extending to the bottom of the hatching vessel. After 2 days of incubation at 23–25 °C, the nauplii were collected using a Pasteur pipette after attracting the organisms to one side of the vessel with a light source. Test compounds were serially diluted 2-fold at a final concentration ranging from 15 to 60 µM (2.5% DMSO, *v*/*v*) in 96-well microplates containing 100 µL of 0.9% NaCl solution with dried yeast (6 mg/L). All experiments were performed in quadruplicate. Vehicle control wells with 2.5% DMSO were included in the experiment as a control for nauplii survival. A 10% DMSO treatment was used as a positive control for mortality. A suspension containing 8–12 nauplii (100 µL) was added to each well, and the plates were incubated at 23–25 °C for 24 h. The numbers of live and dead (non-motile) nauplii in each well were counted under a microscope. Nauplii were considered dead if they remained immobile for 10 s under light exposure. The survival percentage for each test group was calculated relative to that of the vehicle control group, which was set at 100%.

### 2.10. M. tuberculosis Susceptibility Investigation

*M. tuberculosis* H37Ra susceptibility to BD-15 and DG was investigated using a microplate resazurin reduction assay as an indicator of growth [24]. The preparation and dilution of BD-15 and DG followed the protocol described in the *M. smegmatis* susceptibility investigation. Mycobacterial suspensions were cultivated and diluted in 7H9 medium to an optical density (OD_595nm_) of 0.006, and 100 µL was added to each well. After incubation at 37 °C for 7 days, 30 µL of sterile resazurin solution (0.02%) was added to the plates, and the results were evaluated after 48 h. The effects on bacterial viability were considered as the drug concentration that prevented a color change from blue (resazurin) to pink (resorufin) [24].

### 2.11. Evaluation of the Therapeutic Potential of BD-15 in M. tuberculosis-Infected RAW 264.7 Cells

For the *M. tuberculosis* H37Ra infection test, RAW 264.7 cells were seeded in 96-well culture plates at a density of 4 × 10^3^ cells/well in DMEM medium (with 10% FBS, without antibiotics) and incubated overnight at 37 °C with 5% CO_2_ [25]. Infection of RAW 264.7 cells with *M. tuberculosis* was performed at an MOI of 1:1 for 3 h at 37 °C with 5% CO_2_. The medium containing non-internalized bacteria was removed, and the infected cells were treated with BD-15 at a concentration of 10 µM and maintained in culture for 24 or 48 h. After incubation, macrophages were lysed with 0.1% TritonX-100, and the resulting suspensions were serially diluted and plated on Middlebrook 7H10 plus 10% OADC. Plates were incubated for 4 weeks at 37 °C, and the data were expressed as log_10_ (CFUs/well).

### 2.12. Statistical Analysis

Statistical analyses were performed using GraphPad Prism 9.0 (GraphPad Software, San Diego, CA, USA). Data from infection models, *A. salina* survival, and cytotoxicity assays were analyzed using one-way analysis of variance (ANOVA) followed by Dunnett’s post hoc test. Cytokine data were analyzed using one-way analysis of variance (ANOVA) followed by Tukey’s test. Differences were considered statistically significant at *p* < 0.05.

### 2.13. Flow Cytometry Data Analysis

Briefly, macrophages were visualized using size (FSC) and granularity (SSC) parameters, and a gate was applied to the cell population. Macrophages were analyzed by measuring the median fluorescence intensity (MFI) corresponding to antibody labeling. MFI data were normalized to that of the control group, which was set to 100%. The data were evaluated using the FlowJo software version 10.

## 3. Results

### 3.1. Evaluation of the Direct Effects of BD-15 and DG on RAW 264.7 Cell Viability

**Figure 2:**
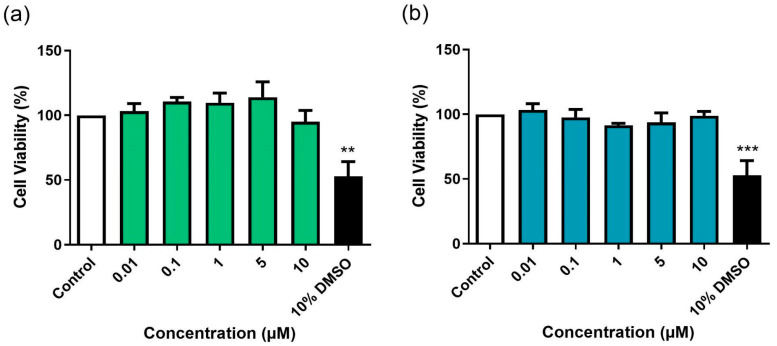
Effects of BD-15 (**a**) and DG (**b**) on RAW 264.7 cell viability after 24 h of incubation. Control: 0.5% DMSO-treated wells, considered 100% cell viability. *** *p* < 0.001 and ** *p* < 0.01 compared to the control. Data were evaluated by ANOVA followed by Dunnett’s post hoc test using GraphPad Prism 9.0.

**Figure 3:**
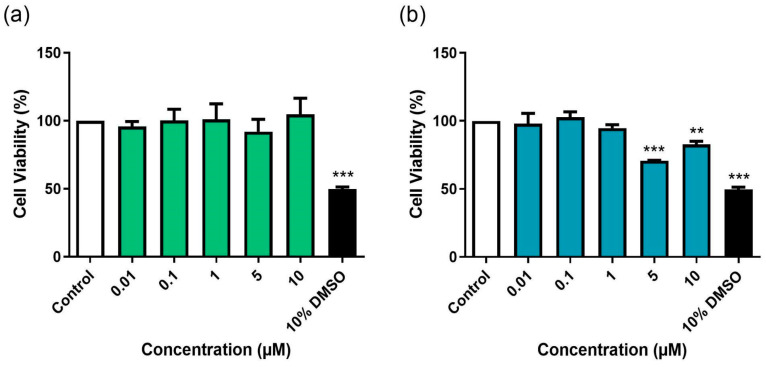
Effects of BD-15 (**a**) and DG (**b**) on RAW 264.7 cell viability after 48 h of incubation. Control: 0.5% DMSO-treated wells, considered 100% cell viability. *** *p* < 0.001 and ** *p* < 0.01 compared to the control. Data were evaluated by ANOVA followed by Dunnett’s post hoc test using GraphPad Prism 9.0.

Prior to infection experiments using macrophages (RAW 264.7 cells), we assessed the direct effects of BD-15 and DG on RAW 264.7 cell viability after 24 h of incubation (Figure 2). Neither BD-15 nor DG affected cell viability at any tested concentration during the 24 h period (Figure 2a,b). Concentrations of BD-15 and DG that maintained macrophage viability above 90% compared to untreated controls after 24 h were further evaluated in 48 h incubation experiments (Figure 3). BD-15 did not affect the cell viability at any of the tested concentrations (Figure 3a). However, DG reduced cell viability at the two highest tested concentrations (5 and 10 μM) (Figure 3b). For subsequent macrophage infection experiments, we selected only concentrations that did not affect cell viability to avoid false-positive results. RAW 264.7 cells treated with 10% DMSO served as positive controls for cell death.

### 3.2. M. smegmatis Susceptibility Investigation and Therapeutic Potential of BD-15 and DG in M. smegmatis-Infected RAW 264.7 Cells

First, we evaluated the effects of BD-15 and DG on the viability of *M. smegmatis* mc^2^155. BD-15 and DG did not affect the viability of *M. smegmatis* in the concentration range of 1.56–200 µM, indicating that their inhibitory concentrations were higher than 200 μM. The minimum inhibitory concentrations found for the positive control drugs used in these experiments, moxifloxacin and rifampicin, were 0.1 and 30 µM, respectively. These findings were observed in two–three independent experiments.

Infection experiments investigated the anti-mycobacterial activity of BD-15 and DG against *M. smegmatis* in RAW 264.7 macrophages. After 24 h of treatment, BD-15 (10 μM) and DG (10 μM) reduced the bacterial burden by 0.266 log_10_ and 0.219 log_10_ CFUs, respectively, compared to the untreated controls (Table 1).

### 3.3. Immunomodulatory Activity of BD-15 and DG During M. smegmatis Infection in RAW 264.7 Cells

**Figure 4:**
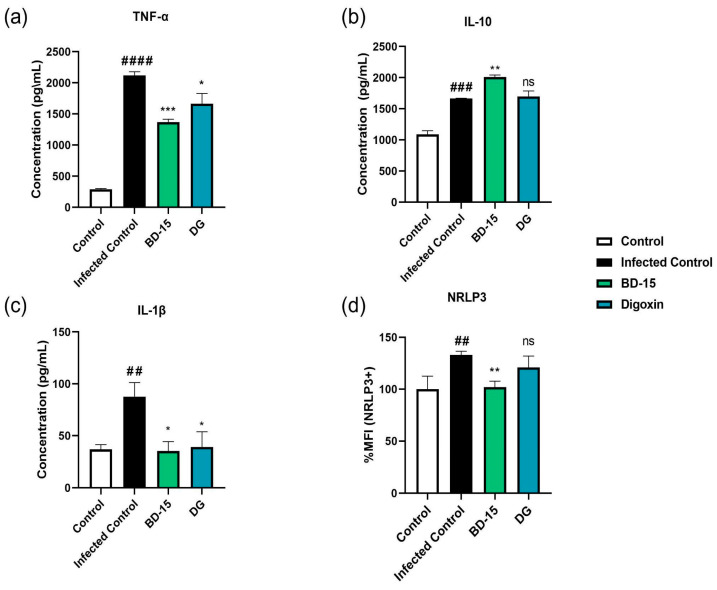
Effects of BD-15 and DG on cytokine levels and inflammasome (NLRP3) expression in RAW 264.7 cells with *M. smegmatis* after 24 h of incubation. Control: RAW 264.7; Infected Control: RAW 264.7 cells infected with *M. smegmatis* (MOI 1:1). BD-15: infected cells treated with BD-15 (10 μM); DG: infected cells treated with DG (10 μM). * *p* < 0.05; ** *p* < 0.01; *** *p* < 0.001 compared to Infected Control; ## *p* < 0.01; ### *p* < 0.001; #### *p* < 0.0001 compared to Control. Data were analyzed by ANOVA followed by Tukey’s post hoc test using GraphPad Prism 9.0. (**a**) TNF-α production in pg/mL; (**b**) IL-10 production in pg/mL; (**c**) IL-1β production in pg/mL; (**d**) percentage of MFI of NLRP3+ cells normalized to control expression.

To assess the immunomodulatory effects of BD-15 and DG, we evaluated the production of cytokines TNF-α, IL-10, and IL-1β in murine macrophages (RAW 264.7) infected with *M. smegmatis*. The results indicated that 24 h post-infection (MOI 1:1), there was a significant increase in the production of TNF-α, IL-1β, and IL-10 compared to that in the control group (Figure 4a–c). Treatment of infected cells with BD-15 (10 μM) resulted in a reduction in TNF-α production by 35.04% and IL-1β production by 59.7%, respectively (Figure 4a,c). Similarly, the DG treatment (10 μM) decreased TNF-α production by 21.42% and IL-1β production by 55.26%, respectively (Figure 4a,c). Regarding IL-10, the BD-15 treatment led to a 20.87% increase in IL-10 levels compared to the infected group, whereas the DG treatment did not show a similar effect (Figure 4b). Additionally, we investigated whether *M. smegmatis* infection in murine macrophages enhances NLRP3 inflammasome expression and whether BD-15 or DG treatment could modulate this response. The data revealed that *M. smegmatis* infection increased NLRP3 expression by 32.9% compared with that in the control group (Figure 4d). In contrast, treatment with BD-15 for 24 h reduced NLRP3 expression by 30.9%, whereas the DG treatment did not show similar effects (Figure 4d).

### 3.4. Cytotoxicity Evaluation of BD-15 and DG on HepG2 Cell Viability and on A. salina Survival

**Figure 5:**
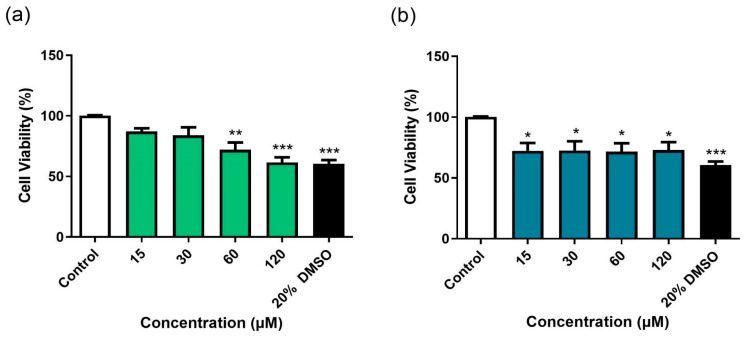
Effects of BD-15 (**a**) and DG (**b**) on HepG2 viability after 24 h of incubation. Control: 1.5% DMSO-treated group, considered 100% cell viability. *** *p* < 0.001, ** *p* < 0.01, and * *p* <0.05 compared with the control group. Data were evaluated by ANOVA followed by Dunnett’s post hoc test using GraphPad Prism 9.0.

After determining the immunomodulatory effects of BD-15 and DG, their cytotoxicity was evaluated in HepG2 cells. Cytotoxicity experiments conducted at concentrations ranging from 15 to 120 µM demonstrated that BD-15 affected cell viability at the two highest concentrations tested (Figure 5a), whereas DG affected cell viability at all concentrations assessed (Figure 5b). DMSO at a concentration of 20% was used as the positive control for the cytotoxicity experiments.

We then estimated that the IC_50_ value for BD-15 would be higher than 120 µM in the HepG2 eukaryotic cell line. The selectivity index was estimated to be higher than 12 based on the concentration of 10 µM used in the efficacy assays (macrophage infections). These results reinforced the potential of BD-15 for further evaluation.

**Figure 6:**
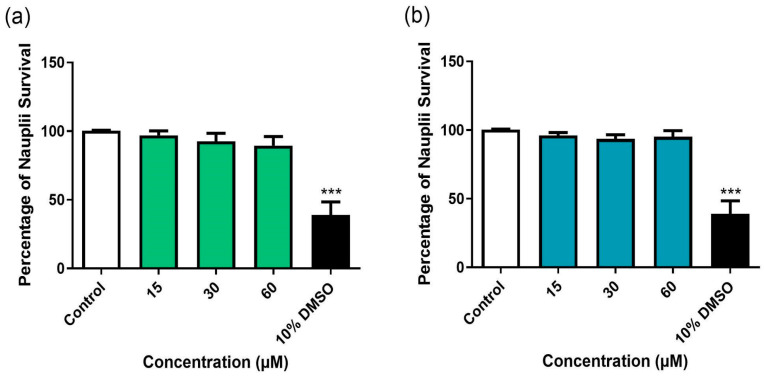
Effects of BD-15 (**a**) and DG (**b**) on *A. salina* survival after 24 h incubation. Control: 2.5% DMSO-treated group, considered 100% *A. salina* survival. *** *p* <0.001 compared to the control group. Data were evaluated by ANOVA followed by Dunnett’s post hoc test using GraphPad Prism 9.0.

BD-15 and DG were evaluated for their potential toxic effects in brine shrimp (*A. salina*). Toxicity experiments conducted across concentration ranges of 15–60 µM demonstrated that BD-15 and DG did not affect nauplii survival at any concentration assessed (Figure 6). The 10% DMSO treatment was used as a positive control for mortality.

### 3.5. M. tuberculosis Susceptibility Investigation and Therapeutic Potential of BD-15 in M. tuberculosis-Infected RAW 264.7 Cells

When tested against M. tuberculosis, BD-15 showed no direct inhibitory effect on bacterial growth over seven days of incubation, indicating that the inhibitory concentration was greater than 200 μM for BD-15. The minimum inhibitory concentrations found for the positive control drugs used in these experiments, moxifloxacin and rifampicin, were 0.2 and 0.03 µM, respectively. These findings were observed in two–three independent experiments.

**Figure 7:**
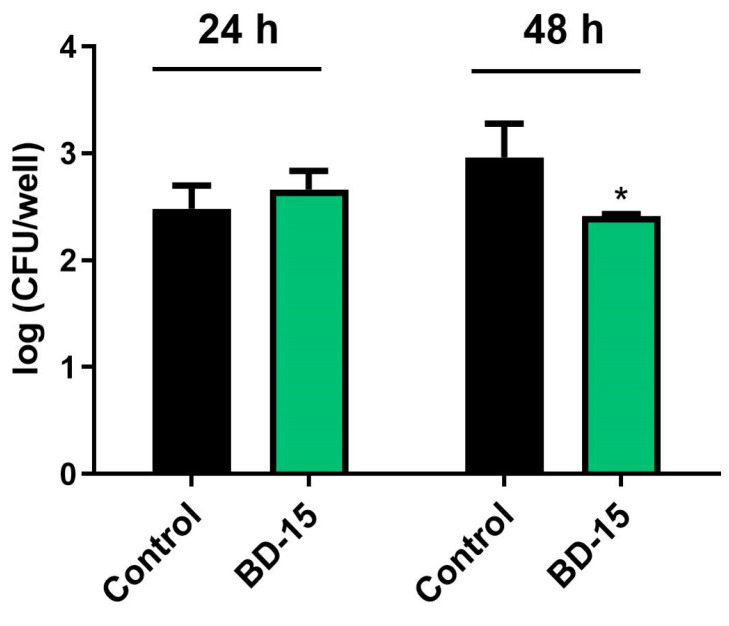
Effects of BD-15 on macrophages infected with Mtb Control: 0.5% DMSO-treated group. * *p* < 0.05 compared with the control group. Data were evaluated using t-tests in GraphPad Prism 9.0.

The infection experiment investigated the effects of BD-15 on M. tuberculosis-infected RAW 264.7 macrophages (Figure 7). Based on previous viability studies of RAW 264.7 cells exposed to BD-15, the treatment duration could be extended to 48 h for infected macrophages. After 48 h of incubation, BD-15 at 10 μM demonstrated an inhibitory effect on intracellular bacterial growth, resulting in a 0.548 log10 decrease in CFU counts compared to the control group (Figure 7).

## 4. Discussion

The immunomodulatory effects of cardiotonic steroids are well established [28,29,30,31,32]. However, the cellular toxicity of this class of molecules must be considered when investigating their therapeutic use [33]. In this study, the synthetic cardiotonic steroid BD-15 was used. This molecule was synthesized via a vinylogous aldol reaction previously described between DG and the appropriate benzaldehyde [17]. It has been demonstrated that DG derivatives obtained through modifications in the steroid lactone ring exhibit lower in vitro cytotoxicity compared to the original molecule [22,34]. In line with this, BD-15 did not reduce the viability of RAW 264.7 cells at the tested times and concentrations (Figure 2a and Figure 3a). However, DG showed cytotoxic effects in this cell line after 48 h of treatment, starting at 5 μM (Figure 3b). It is known that the cytotoxicity of cardiotonic steroids depends on the type of sugar, spatial orientation, and size of their carbohydrate side chain [35,36]. In this regard, adding a bulky side chain to the lactone ring of cardiotonic steroids, as in the synthesis of BD-15, may alter the orientation of the sugar moiety in this molecule. This modification can decrease its affinity for the sodium–potassium pump, thereby reducing its cytotoxicity [22].

The development of infection models with *M. smegmatis* and RAW 264.7 macrophages has been previously described. Li et al. [37] used *M. smegmatis* as a model for mycobacterial infection in macrophages to study the effect of methylprednisolone on mycobacterial proliferation. Bao et al. [38] investigated the effect of cisplatin on mycobacterial proliferation within macrophages by developing a model of *M. smegmatis* infection in RAW 264.7 macrophages. Tests using *M. smegmatis* provide an effective preliminary model for evaluating the activity of drugs against *Mycobacterium* spp. [39]. The complete genome sequence of *M. smegmatis* is available [40], and comparative genomic analyses have shown that it shares numerous genetic features with *M. tuberculosis*, making this microorganism a valuable model for studying the general biology of mycobacteria [41].

In this study, when evaluating the effect of BD-15 and DG directly on *M. smegmatis*, no alteration in bacterial viability was observed at the tested concentrations. These findings can be compared to the study by Mitini-Nkhoma et al. [42], which used a strain of *Mycobacterium bovis* and evaluated the effects of DG at a concentration of 2 nM, observing no bactericidal activity at any tested time point, but they reported bacteriostatic activity [42]. Although no direct reduction in bacterial viability was observed in this study, treatment with DG and BD-15 of *M. smegmatis*-infected RAW 264.7 macrophages resulted in a reduction in the bacterial burden (Table 1). This suggests that the activity of DG and BD-15 in this macrophage murine cell line may have promoted cellular and molecular changes that interfered with bacterial replication.

*M. tuberculosis* infection begins when this mycobacterium is deposited in the lower respiratory tract of individuals and is phagocytosed by resident alveolar macrophages, where it remains for the first 10 days of infection. These alveolar macrophages are the main cell types involved in the initiation of the immune response against *M. tuberculosis* [43]. In this study, the macrophage-like cell line RAW 264.7 was used, which was derived from Balb/c mice injected with Abelson murine leukemia virus. This cell line has been extensively utilized in macrophage research and is considered a good in vitro model for studying responses mediated by this immune cell type [44].

The results demonstrated that infection of RAW 264.7 cells with *M. smegmatis* led to increased production of the pro-inflammatory cytokines TNF-α and IL-1β. It is known that alveolar macrophages infected with *M. tuberculosis* produce a wide range of pro-inflammatory cytokines, such as TNF-α and IL-1β [45,46], and that they may undergo two distinct types of cell death: apoptosis and necrosis. Sustained dysregulated levels of TNF-α can induce necrotic cell death, which is associated with the persistence of *M. tuberculosis* infection, as this type of cell death has been linked to bacterial dissemination [47], promoting the release of viable bacteria from the intracellular to the extracellular environment [48]. Conversely, apoptosis of infected alveolar macrophages contributes to the eradication and restriction of infection [48]. Apoptosis is described as an anti-inflammatory process, as it leads to the production of anti-inflammatory mediators and the inhibition of pro-inflammatory mediators, such as IL-6 and TNF-α [49]. This study showed that treatment of BD-15 and DG in RAW 264.7 cells infected with *M. smegmatis* reduced the production of TNF-α, which may be beneficial for decreasing necrotic cell death in the macrophage population and promoting apoptosis. In the context of mycobacterial infections, apoptosis is advantageous for restricting infection. This relationship may provide an insight into why a reduction in bacterial burden was observed in the population of *M. smegmatis*-infected cells treated with BD-15 and DG.

In addition to TNF-α, the levels of IL-1β were also negatively modulated by treatment with BD-15 and DG in RAW 264.7 cells infected with *M. smegmatis*. Furthermore, treatment with BD-15, but not DG, reduced the expression of NLRP3 inflammasome in infected cells, restoring it to levels observed in the non-infected cell population. The pro-inflammatory cytokine IL-1β exhibits contrasting effects during *M. tuberculosis* infection, with evidence indicating that it can protect the host and increase susceptibility to infection [50]. There is evidence that increased IL-1β expression is associated with more severe TB cases in humans [51]. Moreover, studies using mouse models have demonstrated that suppression of inflammasome activation is part of the antibacterial mechanism of nitric oxide during *M. tuberculosis* infection [52,53]. Supporting this, it has been shown that inflammasome inhibition reduces the survival of *M. tuberculosis* strains in macrophages [54]. Here, treatment with BD-15 not only reduced IL-1β production in the RAW 264.7 population infected with *M. smegmatis* but also led to a decreased expression of the NLRP3 inflammasome. According to what was presented, this could partly explain the reduced bacterial proliferation in RAW 264.7 cells following treatment with BD-15.

Regarding IL-10 production, it is known that during *M. tuberculosis* infection, macrophages are the main producers of this cytokine, which promotes the inhibition of chemokine production by immune cells, leading to the downregulation of cell recruitment to the infection site, thereby limiting tissue damage caused by infection [45,55]. In this study, treatment of *M. smegmatis*-infected RAW 264.7 cells with BD-15 increased IL-10 production, in contrast to the results observed with DG. As IL-10 is an anti-inflammatory cytokine, the ability of BD-15 to upregulate its production suggests an anti-inflammatory mechanism of action. Furthermore, this result highlights the potential of BD-15 compared with DG in modulating inflammatory responses and possibly influencing infection control.

In vitro toxicity screening is a valuable tool for reducing costs in the pharmaceutical industry by analyzing the toxicity of many synthetic compounds. These studies can be conducted prior to animal studies and require only relatively small amounts of the compound (0.2–3 mg). Continuous cell lines, such as established human cancer cell lines, have proven extremely useful for in vitro cytotoxicity studies [56]. In this study, we used HepG2 cells to predict the hepatic toxicity of BD-15 and DG. The HepG2 cell line consists of tumor cells derived from the human liver that retain and express systems associated with the metabolism of endogenous and exogenous substances compared with normal hepatocytes, which justifies its use in studying the metabolism of new drug candidates [57]. In our study, we observed that BD-15 affected cell viability at the two highest concentrations assessed (120 and 60 µM), whereas DG affected cell viability at all concentrations assessed. This is the first study to investigate the hepatotoxic potential of BD-15 in HepG2 cells; however, Schoonen et al. [58] reported that DG was toxic at 10^−6^ M in human HepG2 cells but was non-toxic in H4IIE cells, a rat hepatoma cell line. DG can exert various effects on HepG2 cells, including the induction of RORγ transactivation [59] and the enhancement of cholesterol synthesis by increasing the activity of the 3-hydroxy-3-methylglutaryl-coenzyme A reductase enzyme [60]. Additionally, DG stimulates the apoptosis, senescence, and downregulation of HIF1-α in HepG2 cells [61]. Collectively, these processes may contribute to cell death. The results obtained in this study indicate that BD-15 has the potential to be safely assessed in murine models.

Toxicity assays using *A. salina* are commonly employed in toxicity studies, particularly during the early phases of drug development, to assess the safety of new compounds. These bioassays are valuable for screening potential toxins and evaluating the acute toxicity of substances prior to further in-depth testing [62]. In this study, we observed that BD-15 and DG did not affect the survival of nauplii at any of the concentrations assessed. No studies have investigated the toxic potential of BD-15 in *A. salina*; however, Calleja and Persoone (1993) [63] reported that the combination of DG (12.800 μM) and DMSO did not affect nauplii survival. The toxicological results obtained in this study are the first to demonstrate that BD-15 showed minimal toxicity in the proposed model.

Finally, considering the therapeutic potential of BD-15 observed in this study using an in vitro infection model with *M. smegmatis*, we investigated whether this molecule would also exhibit therapeutic potential during the infection process of *M. tuberculosis*. The effect of BD-15 on bacterial proliferation was evaluated in *M. tuberculosis*-infected RAW 264.7 cells. The results demonstrated that treatment with BD-15 effectively reduced the bacterial burden in *M. tuberculosis*-infected RAW 264.7 cells after 48 h of treatment. The reduction in bacterial proliferation promoted by BD-15 was evident during both *M. smegmatis* and *M. tuberculosis* infection. A possible explanation for this finding is that *M. tuberculosis* and *M. smegmatis* share significant genomic similarities [41]. This suggests that the immunomodulatory effects of BD-15 observed during *M. smegmatis* infection of RAW 264.7 cells might also occur during *M. tuberculosis* infection, highlighting the potential of this molecule as a therapeutic agent against *M. tuberculosis*.

**Figure 8:**
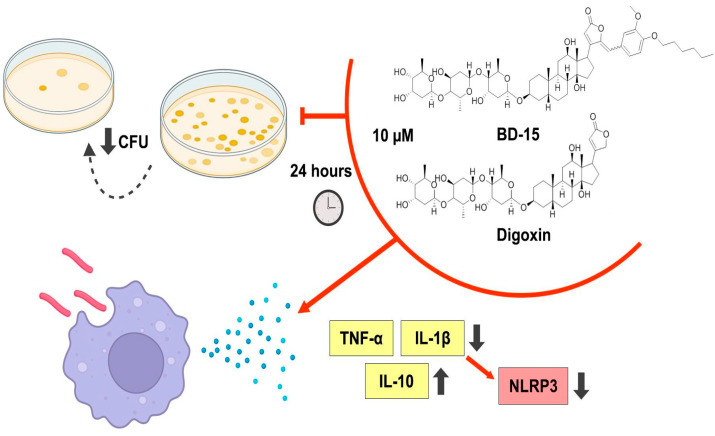
Summary of the effects of BD-15 in *M. smegmatis*-infected RAW 264.7 cells.

In summary, our data have shown that treatment with BD-15 of *M. smegmatis*-infected RAW 264.7 cells not only reduced bacterial proliferation but also exerted immunomodulatory effects, reducing the production of pro-inflammatory cytokines TNF-α and IL-1β and increasing the production of the anti-inflammatory cytokine IL-10, suggesting a possible anti-inflammatory activity for this molecule during mycobacteria infection. In addition, the reduction in IL-1β production promoted by BD-15 was accompanied by a lower expression of the NLRP3 inflammasome, indicating a possible mechanism by which BD-15 may exert its effects (Figure 8).

## 5. Conclusions

The data presented here demonstrate, for the first time, that treatment with the synthetic cardiotonic steroid BD-15 reduces the bacterial burden in both *M. smegmatis*- and *M. tuberculosis*-infected RAW 264.7 cells. The reduction observed during *M. smegmatis* infection was accompanied by decreased production of the pro-inflammatory cytokines IL-1β and TNF-α, reduced expression of the NLRP3 inflammasome, and increased production of IL-10. Furthermore, the toxicity of this molecule was assessed, and safe concentrations were established. These findings contribute to the understanding of the potential of BD-15 in combating infections caused by mycobacteria and open opportunities to explore its activity in different immunological contexts. However, further studies are necessary to gain a deeper understanding of the cellular and molecular modifications induced by BD-15 during mycobacterial infections. Therefore, the use of additional infection models, both in vitro and in vivo, is essential.

## 6. Limitations

Although the study revealed the immunomodulatory and anti-mycobacterial potential of BD-15, some important limitations should be considered. There are still no pharmacokinetic (PK) data for the compound, so we do not know whether it reaches the 10 µM concentration considered relevant when administered orally (PO) or intraperitoneally (IP). Furthermore, the experiments were performed exclusively in in vitro models, limiting the extrapolation of the results to more complex in vivo systems. However, studies with animal models are ongoing to evaluate the bioavailability, plasma levels, and efficacy of BD-15 in biological conditions closer to the clinical context, which will help to overcome these gaps.

## Figures and Tables

**Table 1:** Intracellular effects of BD-15 and DG in macrophages infected with *M. smegmatis*.

Compound (Concentration in µM)	Mean log_10_ CFUs/Well ± SD	Compound (Concentration in µM)	Mean log_10_ CFUs/Well ± SD
Untreated	6.31 ± 0.15	Untreated	6.04 ± 0.25
BD-15 (1)	6.30 ± 0.05	BD-15 (0.01)	6.09 ± 0.21
BD-15 (10)	6.05 ± 0.07 *	BD-15 (0.1)	6.12 ± 0.14
DG (1)	6.17 ± 0.14	DG (0.01)	6.11 ± 0.06
DG (10)	6.09 ± 0.01 *	DG (0.1)	6.08 ± 0.10

* *p <* 0.05 compared to the untreated wells.

## Data Availability

The data used to support the findings of this study are available from the corresponding authors upon request.

## Supplementary Materials

The following supporting information can be downloaded at: https://www.mdpi.com/article/10.3390/microorganisms13020269/s1, Figure S1: HPLC analysis of BD-15; Figure S2: ^1^H NMR (400 MHz-CDCl_3_) spectrum of compound BD-15; Figure S3: ^13^C NMR (100 MHz-CDCl_3_) spectrum of compound BD-15; Figure S4: ESI MS [-] Analysis of BD15; Figure S5: ESI MS [+] analysis of BD15; Figure S6: Infrared spectrum of compound BD-15.
