# Supplementary material for: Evaluation of the Anti-Mycobacterial and Anti-Inflammatory Activities of the New Cardiotonic Steroid γ-Benzylidene Digoxin-15 in Macrophage Models of Infection
Source: Microorganisms. 2025 Jan 25;13(2):269. doi: 10.3390/microorganisms13020269 (PMC11857721; doi:10.3390/microorganisms13020269)
Supplement: Supplementary file 1 [file microorganisms-13-00269-s001.zip › Figure S1 HPLC analysis of BD-15.pdf]

Figure S1. HPLC analysis of BD-15

==== Shimadzu LCsolution Analysis Report ====

C:\LabSolutions\Data\Ze\Ericke\BD's coluna nova\BD-15 purificada19.lcd

Acquired by	: Admin
Sample Name	: BD-15 purificada19
Sample ID	: Ericke
Vail #	:
Injection Volume	: 20 uL
Data File Name	: BD-15 purificada19.lcd
Method File Name	: metodo_C18_MeOH_H2O_70-30.lcm
Batch File Name	:
Report File Name	: Default.lcr
Data Acquired	: 23/06/2024 17:54:09
Data Processed	: 23/06/2024 18:25:04

<Chromatogram>

C:\LabSolutions\Data\Ze\Ericke\BD's coluna nova\BD-15 purificada19.lcd

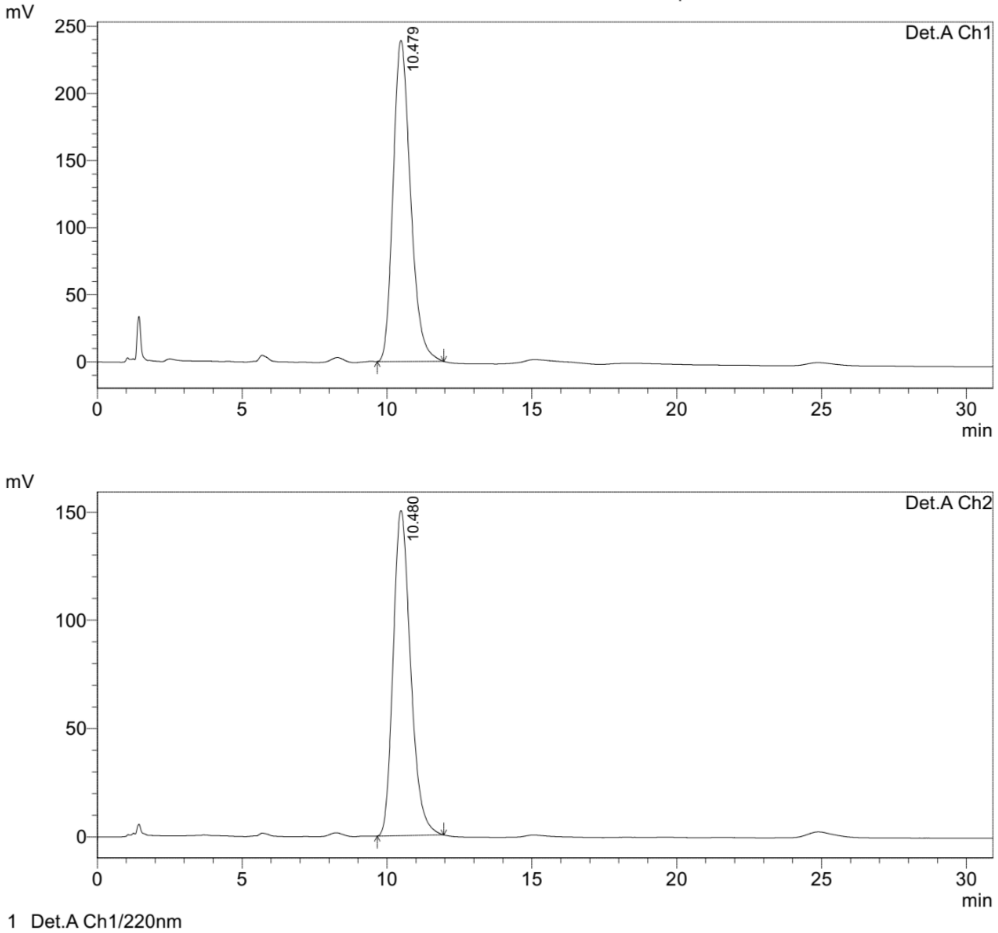

1 Det.A Ch1/220nm
2 Det.A Ch2/254nm

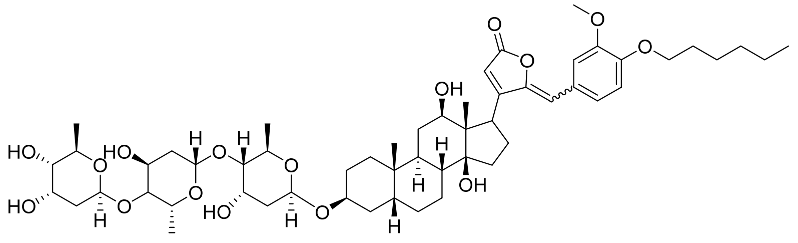

BD15

$C_{55}H_{82}O_{16}$

M.M.: 999,23 $g.mol^{-1}$

^{1}H RMN (400 MHz, $CDCl_3$) δ (ppm): 0.73 (s, 3H); 0.75 (s, 3H); 0.94 – 0.83 (m, 9H); 1.84 – 1.03 (m, 61H); 2.01 – 1.88 (m, 2H); 2.21 – 2.08 (m, 10H); 2.99 (s, 2H); 3.06 (s, 2H); 3.33 – 3.17 (m, 6H); 3.39 (td, *J* = 8.6, 3.1 Hz, 2H), 3.56 (dd, *J* = 11.4, 5.8 Hz, 2H); 3.83 (s, 3H); 3.90 (s, 1H); 3.95 (d, *J* = 1.4 Hz, 2H); 3.97 (s, 2H); 3.99 (s, 1H); 4.02 (s, 2H); 4.11 (t, *J* = 3.2 Hz, 2H); 4.24 (dt, *J* = 6.6, 3.3 Hz, 4H); 4.95 – 4.81 (m, 6H); 6.68 – 6.58 (m, 4H); 6.81 (dd, *J* = 8.3, 1.8 Hz, 2H). 7.25 (s, 1H); 7.27 (s, 1H).

^{13}C RMN (100 MHz, $CDCl_3$) δ (ppm): 204.62, 204.39, 200.49, 200.27, 168.71, 168.35, 149.75, 148.47, 148.43, 146.59, 146.41, 124.73, 124.38, 120.81, 120.60, 113.19, 113.17, 112.21, 111.85, 98.28, 98.21, 95.43, 86.65, 86.62, 82.56, 82.21, 77.35, 77.23, 77.03, 76.71, 72.72, 72.53, 69.49, 69.07, 68.26, 68.10, 66.46, 66.37, 56.43, 56.35, 56.30, 56.09, 55.98, 42.86, 42.52, 41.76, 41.68, 37.82, 37.13, 36.71, 36.24, 35.04, 33.24, 32.25, 31.57, 30.15, 29.81, 29.24, 29.13, 29.05, 28.07, 27.59, 26.63, 26.49, 25.61, 23.56, 23.53, 22.59, 21.81, 18.18, 18.16, 14.03, 9.31, 9.13.

IV ($\bar{\upsilon}_{max}$, KBr, cm^{-1}): 3474 (O-H); 2930, 2874 (C-H); 1734 (C=O); 1695 (C=O); 1599 (C=C); 1450, 1379 ($-CH_3$); 1256 (C-O-H); 1163, 1070 (C-O); 868 (=C-H).
