# Supplementary material for: Evaluation of the Anti-Mycobacterial and Anti-Inflammatory Activities of the New Cardiotonic Steroid γ-Benzylidene Digoxin-15 in Macrophage Models of Infection
Source: Microorganisms. 2025 Jan 25;13(2):269. doi: 10.3390/microorganisms13020269 (PMC11857721; doi:10.3390/microorganisms13020269)
Supplement: Supplementary file 1 [file microorganisms-13-00269-s001.zip › Figure S2 1H NMR (400 MHz-CDCl3) spectrum of compound BD-15..pdf]

Figure S2. ^1H NMR (400 MHz-$CDCl_3$) spectrum of compound **BD-15**.

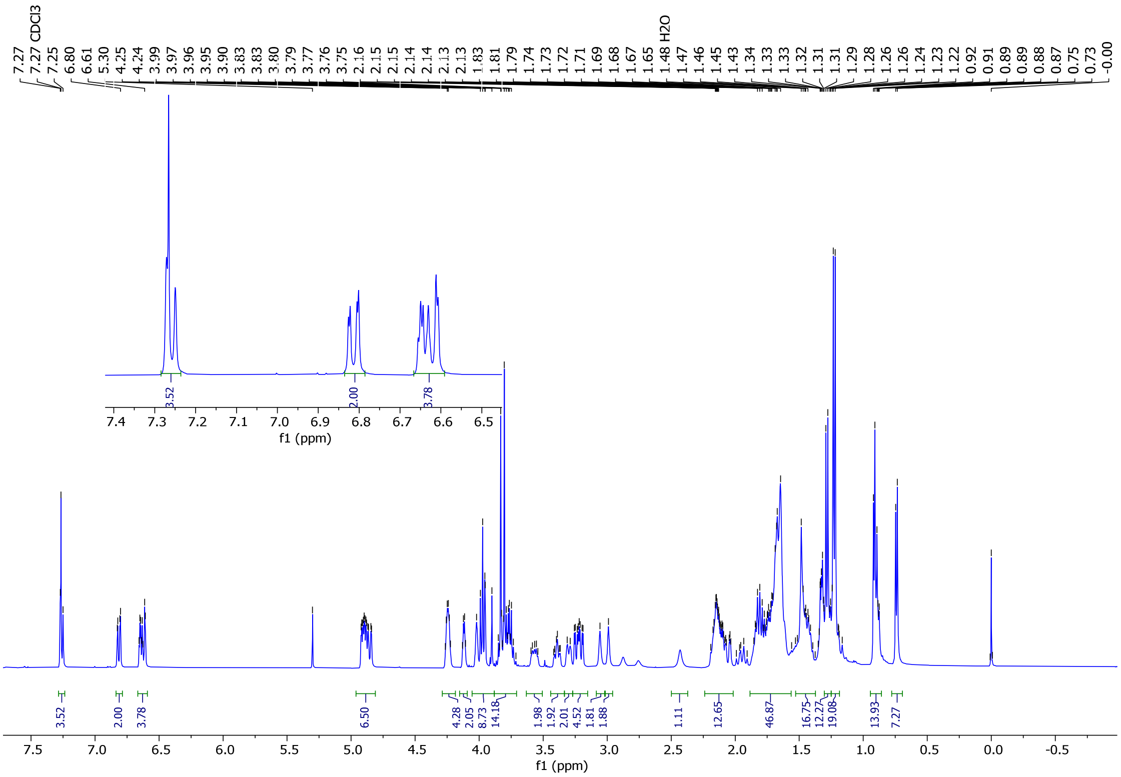

^{1}H RMN (400 MHz, $CDCl_3$) δ (ppm): 0.73 (s, 3H); 0.75 (s, 3H); 0.94 – 0.83 (m, 9H); 1.84 – 1.03 (m, 61H); 2.01 – 1.88 (m, 2H); 2.21 – 2.08 (m, 10H); 2.99 (s, 2H); 3.06 (s, 2H); 3.33 – 3.17 (m, 6H); 3.39 (td, *J* = 8.6, 3.1 Hz, 2H), 3.56 (dd, *J* = 11.4, 5.8 Hz, 2H); 3.83 (s, 3H); 3.90 (s, 1H); 3.95 (d, *J* = 1.4 Hz, 2H); 3.97 (s, 2H); 3.99 (s, 1H); 4.02 (s, 2H); 4.11 (t, *J* = 3.2 Hz, 2H); 4.24 (dt, *J* = 6.6, 3.3 Hz, 4H); 4.95 – 4.81 (m, 6H); 6.68 – 6.58 (m, 4H); 6.81 (dd, *J* = 8.3, 1.8 Hz, 2H); 7.25 (s, 1H); 7.27 (s, 1H).
