# Supplementary material for: Evaluation of the Anti-Mycobacterial and Anti-Inflammatory Activities of the New Cardiotonic Steroid γ-Benzylidene Digoxin-15 in Macrophage Models of Infection
Source: Microorganisms. 2025 Jan 25;13(2):269. doi: 10.3390/microorganisms13020269 (PMC11857721; doi:10.3390/microorganisms13020269)

**Figure S5.** ESI MS [+] Analysis of BD15

(ACN:H2O)

m/z calculated for $C_{55}H_{82}O_{16}$ $[M+Na]^+$: 1021.55, found 1021.52.

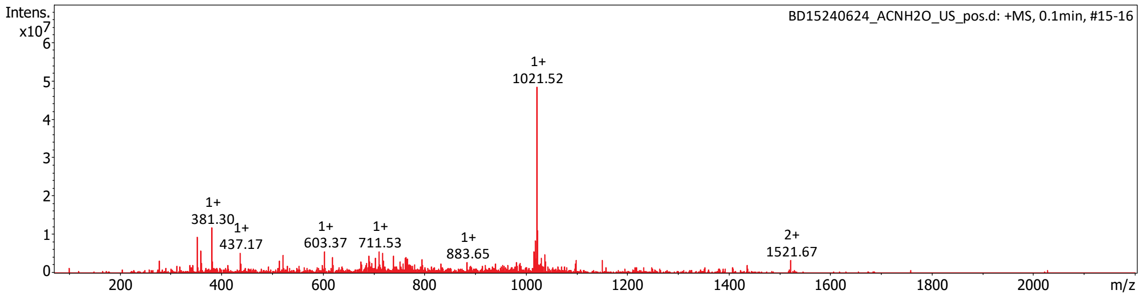

Supplement: Supplementary file 1 [file microorganisms-13-00269-s001.zip › Figure S5 ESI MS [+] Analysis of BD15.pdf]
