# Supplementary material for: Evaluation of the Anti-Mycobacterial and Anti-Inflammatory Activities of the New Cardiotonic Steroid γ-Benzylidene Digoxin-15 in Macrophage Models of Infection
Source: Microorganisms. 2025 Jan 25;13(2):269. doi: 10.3390/microorganisms13020269 (PMC11857721; doi:10.3390/microorganisms13020269)
Supplement: Supplementary file 1 [file microorganisms-13-00269-s001.zip › Figure S6 Infrared spectrum of compound BD-15..pdf]

**Figure S6.** Infrared spectrum of compound **BD-15**.

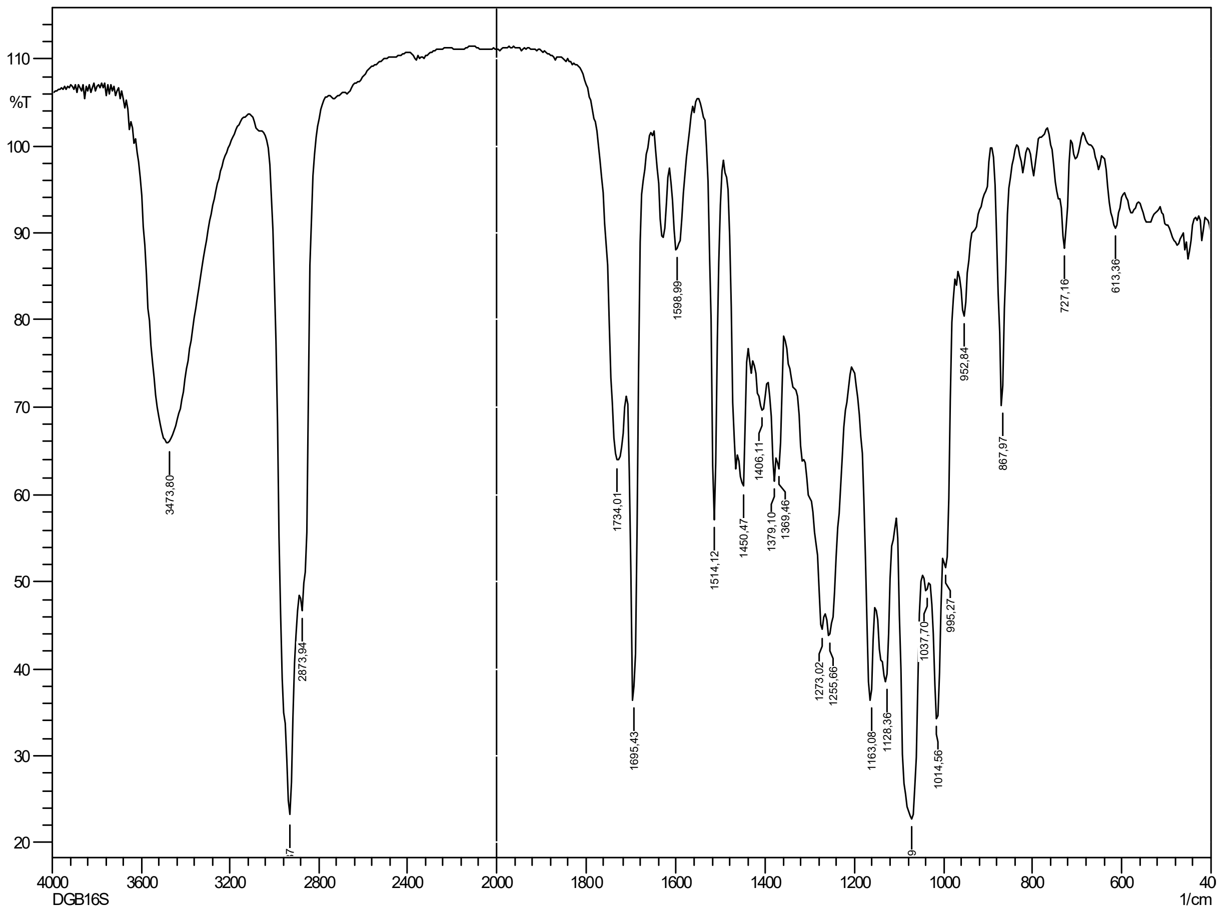


| | Peak | Intensity | Corr. Intensity | Base (H) | Base (L) | Area | Corr. Area |
|---|---|---|---|---|---|---|---|
| 1 | 613,36 | 90,466 | 5,871 | 640,37 | 594,08 | 1,37 | 0,669 |
| 2 | 727,16 | 88,258 | 12,518 | 763,81 | 713,66 | 1,104 | 1,343 |
| 3 | 867,97 | 70,147 | 29,775 | 891,11 | 835,18 | 2,736 | 2,725 |
| 4 | 952,84 | 80,377 | 7,001 | 964,41 | 906,54 | 3,142 | 0,551 |
| 5 | 995,27 | 51,625 | 3,322 | 997,2 | 974,05 | 4,087 | 0,368 |
| 6 | 1014,56 | 34,291 | 16,879 | 1029,99 | 999,13 | 11,384 | 2,423 |
| 7 | 1037,7 | 49,038 | 1,271 | 1045,42 | 1031,92 | 4,111 | 0,082 |
| 8 | 1070,49 | 22,722 | 30,588 | 1103,28 | 1047,35 | 27,859 | 12,729 |
| 9 | 1128,36 | 38,443 | 6,519 | 1136,07 | 1105,21 | 10,025 | 0,62 |
| 10 | 1163,08 | 36,303 | 15,762 | 1203,58 | 1153,43 | 13,138 | 1,959 |
| 11 | 1255,66 | 43,836 | 6,158 | 1263,37 | 1205,51 | 13,921 | 1,071 |
| 12 | 1273,02 | 44,33 | 5,014 | 1311,59 | 1265,3 | 12,497 | 0,392 |
| 13 | 1369,46 | 63,02 | 4,636 | 1373,32 | 1357,89 | 2,523 | 0,275 |
| 14 | 1379,1 | 61,601 | 4,253 | 1392,61 | 1375,25 | 3,157 | 0,226 |
| 15 | 1406,11 | 69,533 | 4,211 | 1425,4 | 1394,53 | 4,495 | 0,453 |
| 16 | 1450,47 | 60,823 | 9,243 | 1460,11 | 1438,9 | 3,855 | 0,686 |
| 17 | 1514,12 | 57,105 | 43,602 | 1546,91 | 1494,83 | 3,523 | 3,854 |
| 18 | 1598,99 | 88,127 | 11,281 | 1614,42 | 1560,41 | 1,196 | 1,399 |
| 19 | 1695,43 | 36,454 | 43,383 | 1710,86 | 1656,85 | 8,166 | 4,873 |
| 20 | 1734,01 | 63,946 | 14,64 | 1820,8 | 1712,79 | 5,919 | 2,028 |
| 21 | 2873,94 | 46,691 | 5,26 | 2881,65 | 2758,21 | 10,43 | 0,878 |
| 22 | 2929,87 | 22,865 | 40,274 | 3051,39 | 2883,58 | 45,577 | 21,211 |
| 23 | 3473,8 | 65,87 | 0,242 | 3475,73 | 3118,9 | 22,316 | 0,305 |
