# Supplementary figures and images for: Evaluation of the Anti-Mycobacterial and Anti-Inflammatory Activities of the New Cardiotonic Steroid γ-Benzylidene Digoxin-15 in Macrophage Models of Infection
Source: Microorganisms. 2025 Jan 25;13(2):269. doi: 10.3390/microorganisms13020269 (PMC11857721; doi:10.3390/microorganisms13020269)

**Figure S3.**  $^{13}\text{C}$  NMR (100 MHz- $\text{CDCl}_3$ ) spectrum of compound **BD-15**.

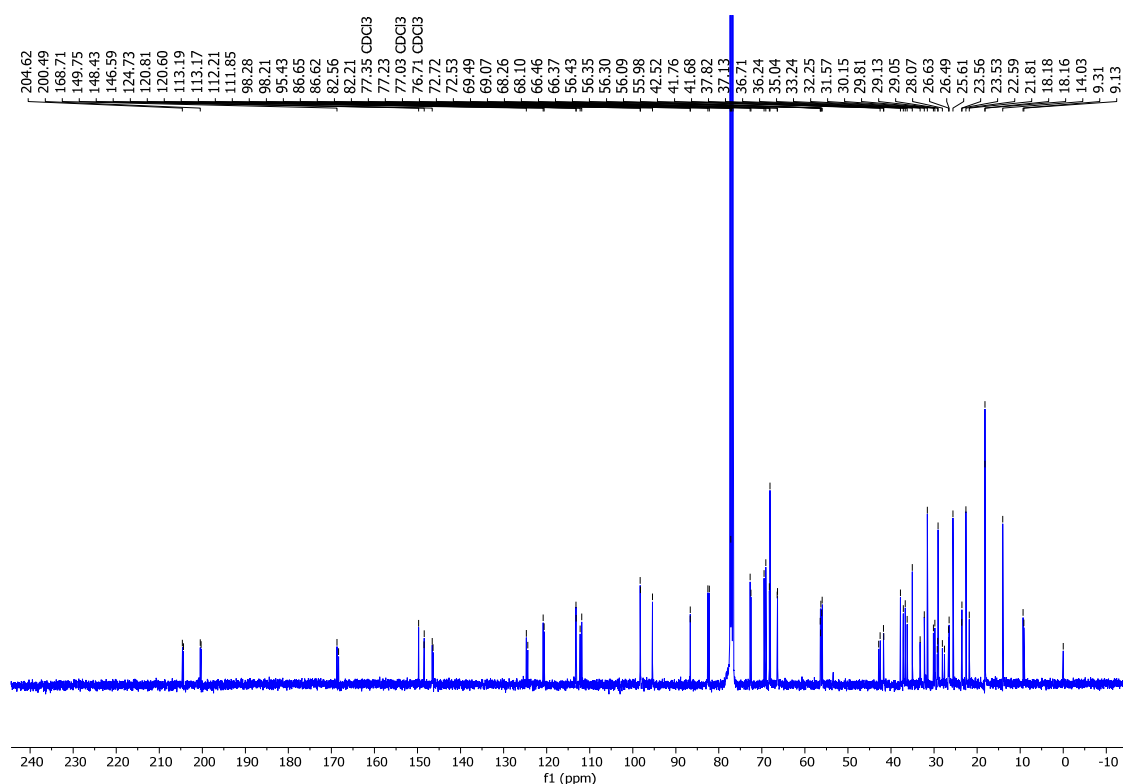

$^{13}\text{C}$  RMN (100 MHz,  $\text{CDCl}_3$ )  $\delta$  (ppm): 204.62, 204.39, 200.49, 200.27, 168.71, 168.35, 149.75, 148.47, 148.43, 146.59, 146.41, 124.73, 124.38, 120.81, 120.60, 113.19, 113.17, 112.21, 111.85, 98.28, 98.21, 95.43, 86.65, 86.62, 82.56, 82.21, 77.35, 77.23, 77.03, 76.71, 72.72, 72.53, 69.49, 69.07, 68.26, 68.10, 66.46, 66.37, 56.43, 56.35, 56.30, 56.09, 55.98, 42.86, 42.52, 41.76, 41.68, 37.82, 37.13, 36.71, 36.24, 35.04, 33.24, 32.25, 31.57, 30.15, 29.81, 29.24, 29.13, 29.05, 28.07, 27.59, 26.63, 26.49, 25.61, 23.56, 23.53, 22.59, 21.81, 18.18, 18.16, 14.03, 9.31, 9.13.

Supplement: Supplementary file 1 [file microorganisms-13-00269-s001.zip › Figure S3 13C NMR (100 MHz-CDCl3) spectrum of compound BD-15..pdf]

**Figure S4.** ESI MS [-] Analysis of BD15 (ACN:H2O)

**ESI MS [-]** (ACN:H2O)

m/z calculated for  $C_{55}H_{82}O_{16}[M-H]^-$ : 997.55, found 997.83.

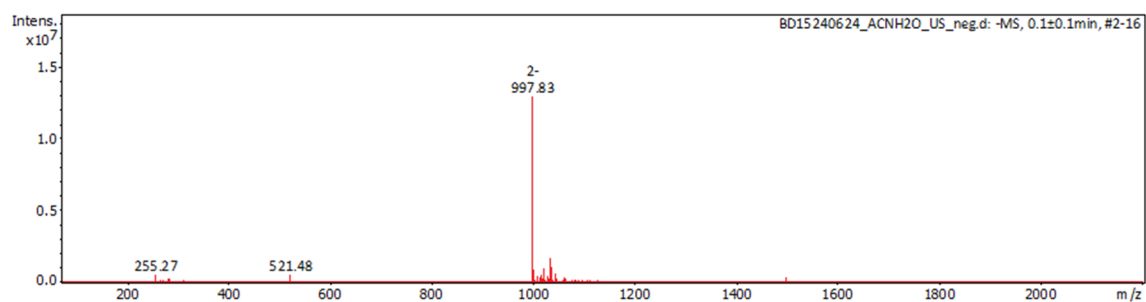

Supplement: Supplementary file 1 [file microorganisms-13-00269-s001.zip › Figure S4 ESI MS [-] Analysis of BD15.pdf]
